# Cleavage Off-Loading and Post-assembly-Line Conversions Yield Products with Unusual Termini during Biosynthesis

**DOI:** 10.1021/acschembio.2c00367

**Published:** 2022-07-21

**Authors:** Yi-Ming Shi, Merle Hirschmann, Yan-Ni Shi, Helge B. Bode

**Affiliations:** † Department of Natural Products in Organismic Interactions, Max Planck Institute for Terrestrial Microbiology, 35043 Marburg, Germany; ‡ Molecular Biotechnology, Department of Biosciences, Goethe University Frankfurt, 60438 Frankfurt am Main, Germany; § Chemical Biology, Department of Chemistry, Philipps University Marburg, 35043 Marburg, Germany; ∥ Senckenberg Gesellschaft für Naturforschung, 60325 Frankfurt am Main, Germany

## Abstract

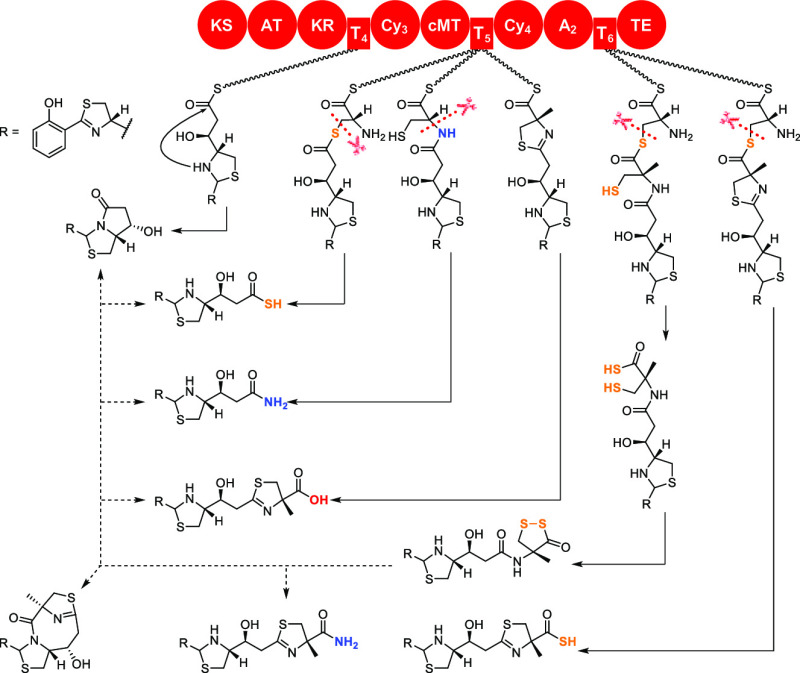

Piscibactins and photoxenobactins are metallophores and virulence factors, whose biosynthetic gene cluster, termed *pxb*, is the most prevalent polyketide synthase/non-ribosomal peptide synthetase hybrid cluster across entomopathogenic bacteria. They are structurally similar to yersiniabactin, which contributes to the virulence of the human pathogen *Yersinia pestis*. However, the *pxb*-derived products feature various chain lengths and unusual carboxamide, thiocarboxylic acid, and dithioperoxoate termini, which are rarely found in thiotemplated biosyntheses. Here, we characterize the *pxb* biosynthetic logic by gene deletions, site-directed mutagenesis, and isotope labeling experiments. Notably, we propose that it involves (1) heterocyclization domains with various catalytic efficiencies catalyzing thiazoline and amide/thioester bond formation and (2) putative C–N and C–S bond cleavage off-loading manners, which lead to products with different chain lengths and usual termini. Additionally, the post-assembly-line spontaneous conversions of the biosynthetic end product contribute to production titers of the other products in the culture medium. This study broadens our knowledge of thiotemplated biosynthesis and how bacterial host generate a chemical arsenal.

## Introduction

**Figure 1:**
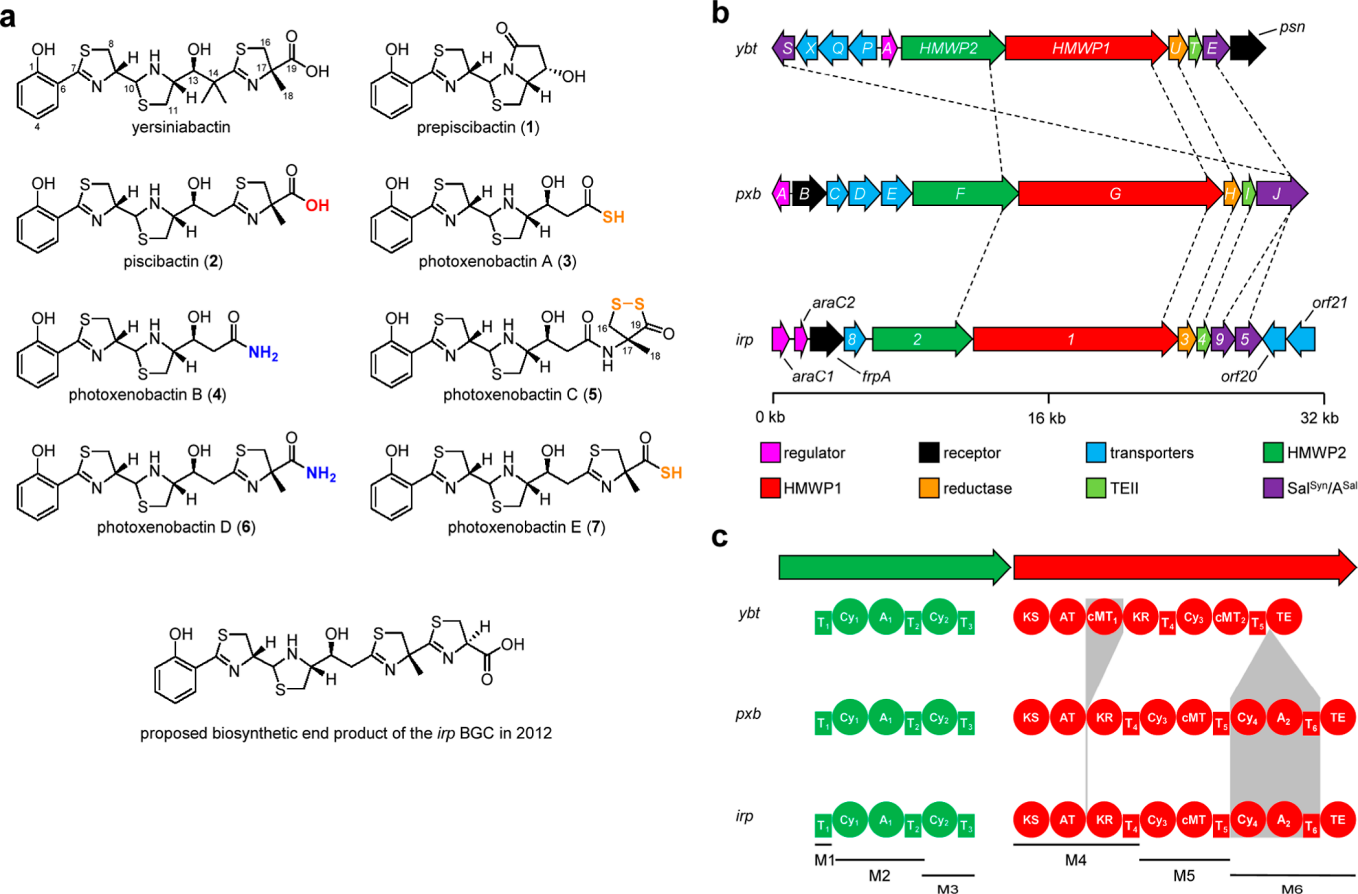
Chemical structures of yersiniabactin and yersiniabactin-like natural products and BGCs encoding their biosyntheses. (a) Chemical structures of yersiniabactin from *Y. pestis*, *pxb*-derived products (**1**–**7**) from *X. szentirmaii* DSM 16338, and the proposed biosynthetic end product^7^ of the *irp* BGC from *P. damselae* subsp. *piscida*. The terminal heteroatoms are highlighted. (b) Comparison of yersiniabactin-related BGCs in *Y. pestis* (*ybt*), *X. szentirmaii* (*pxb*), and *P. damselae* subsp. *piscida* (*irp*). Homologous biosynthetic genes between three BGCs are connected with dashed lines. kb, kilobase. (c) Domain organization of HMWP1 and HMWP2 homologues encoded by three BGCs. Domain differences are indicated by shapes in gray. T, thiolation; A, adenylation; Cy, heterocyclization; KS, ketosynthase; AT, acyltransferase; KR, ketoreductase; cMT, carbon methyltransferase; and TE, thioesterase domains.

Recently, we identified *pxb*, a biosynthetic gene cluster (BGC) coding for the most widespread polyketide synthase (PKS)/non-ribosomal peptide synthetase (NRPS) hybrid assembly line in entomopathogenic bacteria *Xenorhabdus* and *Photorhabdus*. By homologous overexpression, we demonstrated that the *pxb* BGC encodes the biosynthesis of an array of yersiniabactin-like natural products, piscibactins (**1** and **2**) and photoxenobactins (**3**–**7**).^1^ Labeling studies revealed that salicylic acid, cysteine, and the methyl moiety of S-adenosyl methionine are the major building blocks.^1^ It is particularly noteworthy for their unusual termini, such as carboxamide, thiocarboxylic acid, and dithioperoxoate (Figure 1a), which are rarely found in thiotemplated natural product biosyntheses.^2,3^ Besides scavenging environmental metal ions (Ga^3+^, Cu^2+^, and Fe^3+^) for the bacterial host, these compounds serve as virulence factors against insects^1^ and thereby facilitate the symbiotic nematodes killing insect prey.^4^

AntiSMASH^5^ analysis revealed that the *pxb* BGC, exemplified by the cluster in *Xenorhabdus szentirmaii* DSM 16338, is similar to both the *ybt* BGC from *Yersinia pestis*(6) and the *irp* BGC from *Photobacterium damselae* subsp. *piscida*(7) (Figure 1b). All *pxb* biosynthetic genes have a counterpart in the *ybt* BGC, while the major differences lie in gene and domain architectures. A bifunctional enzyme (PxbJ) encoded by the *pxb* BGC is equivalent to the salicylate synthase (YbtS) and the free-standing salicylate specific A domain (YbtE) encoded by the *ybt* BGC. Although PxbG and Irp1 are homologous to HMWP1, both PxbG and Irp1 are deficient in carbon methyltransferase (cMT_1_)^8^ specific for C-14 bismethylation of yersiniabactin and encode an additional heterocyclization–adenylation–thiolation (Cy_4_–A_2_–T_6_) module (M6; Figure 1c). A previous report proposed the biosynthesis of prepiscibactin (**1**) and piscibactin (**2**) encoded by the *irp* BGC and hypothesized that the additional Cy_4_–A_2_–T_6_ module might introduce the fourth thiazoline to piscibactin (**2**), resulting in a full biosynthetic product.^7^ However, the newly identified photoxenobactins (**3**–**7**) from the *pxb* BGC featuring various chain lengths and termini defy such a functional prediction of the biosynthetic assembly line,^1^ thus motivating us to characterize the biosynthetic logic of photoxenobactins. Here, we investigate the biosynthesis of photoxenobactins in the overexpressing mutant *X. szentirmaii* P_*BAD*_*pxbF*. Since **7** is only present in the *X. szentirmaii* P_*BAD*_*pxbF* Δ*hfq* mutant^1^ (*hfq* is a global regulator positively affecting secondary metabolism^9^) but is absent in the *X. szentirmaii* P_*BAD*_*pxbF* mutant, **7** is therefore not examined herein.

## Results and Discussion

### Premature Off-Loading of *pxb*-Derived Products

**Figure 2:**
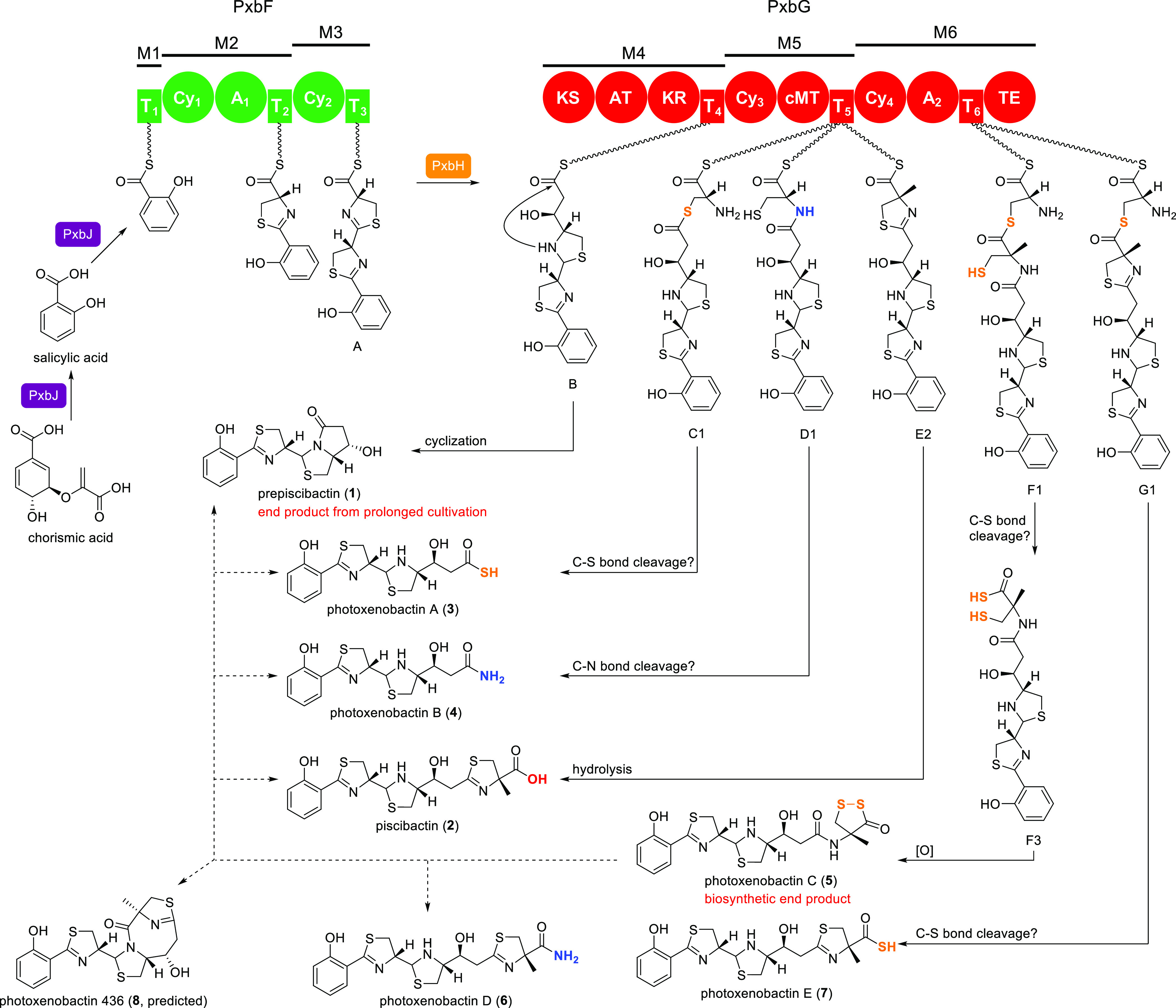
Biosynthetic pathway and post-assembly-line conversions of piscibactins and photoxenobactins in *X. szentirmaii*. Solid and dashed arrows indicate assembly line biosynthesis and post-assembly-line conversions in culture media, respectively. Post-assembly-line conversions from **5** to **1**–**4** and **6** were observed both in vivo and in vitro, while the conversion from **5** to **8** was observed in vitro (Figure 6, traces ii–v).

**Figure 3:**
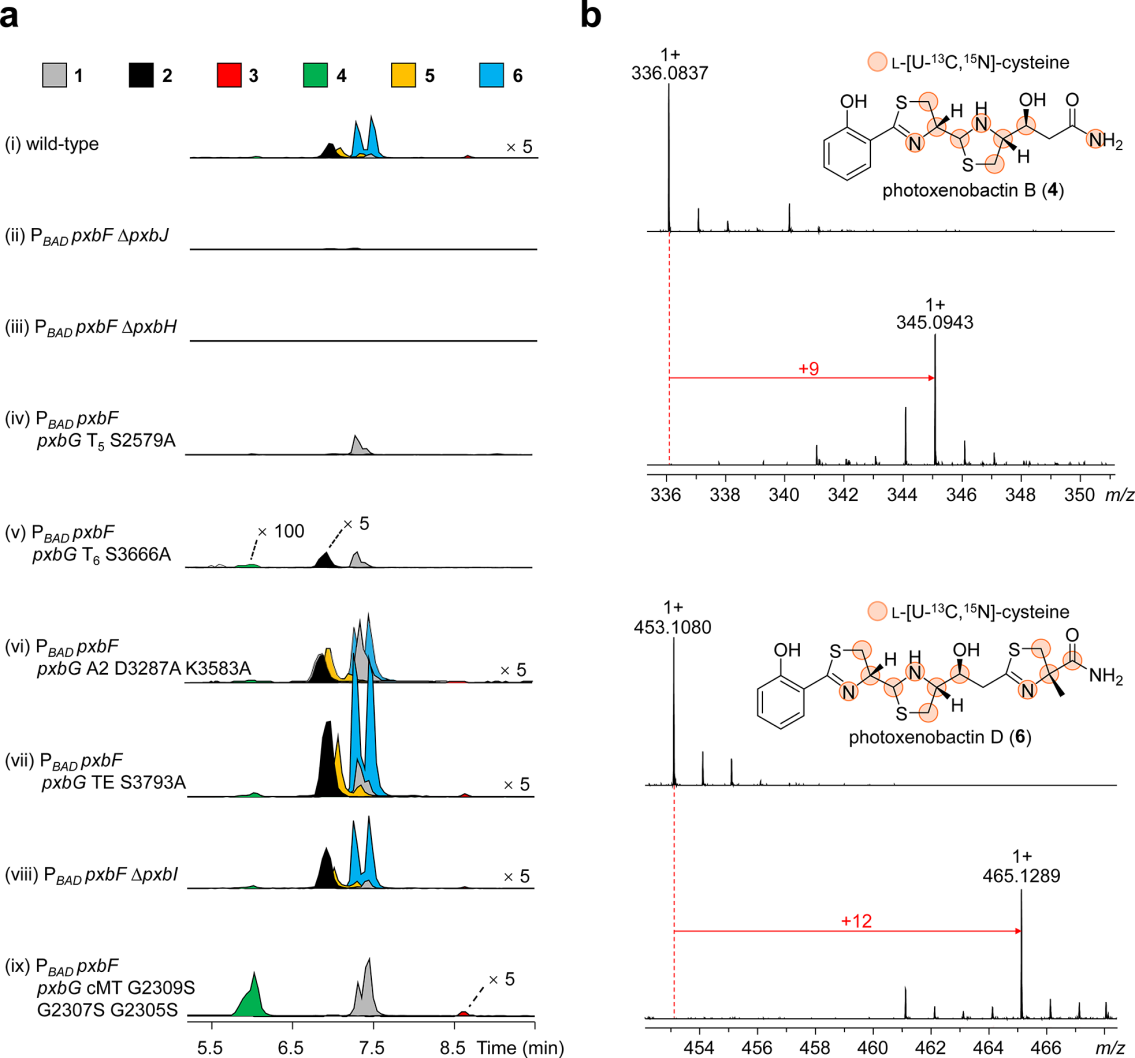
HPLC–MS analysis of the constructed mutants in Sf-900 media and isotope labeling experiments for carboxamide identification. (a) Mutations and deletions were carried out in *X. szentirmaii* P_*BAD*_*pxbF*, which is an overexpressing mutant. Shown are the EICs of piscibactins and photoxenobactins. Each compound contains a pair of C-10 epimers, which results from the thiazolinyl–S–T_3_ reduction with PxbH. Intensities of EICs in traces i and v–ix are magnified for visualizing tiny peaks. Magnifications are indicated on the right side of traces or on the top of the peak. (b) Mass spectrometry identification of nitrogen source of the carboxamide in photoxenobactins B (**4**) and D (**6**) by isotope labeling experiments. Positions shown as colored spheres are incorporated with l-[U–^13^C,^15^N]-cysteine. Parent ions (M–H_2_O + H^+^) are shown. Red arrows indicate mass shifts.

The early-stage biosynthesis of photoxenobactins, until the reduction of intermediate A catalyzed by PxbH (homologous to YbtU, a thiazolinyl–S–T reductase^10^), is postulated to be identical to that of yersiniabactin (Figure 2), which has already been extensively delineated.^6,10^ This is supported by *pxbJ* (encoding a bifunctional salicylate synthase/adenylation protein) and *pxbH* deletion mutants, both of which abolished production of **1**–**6** (Figure 3a traces ii and iii), and complementation thereof that restored their production to original levels (Figure S1).

**Figure 4:**
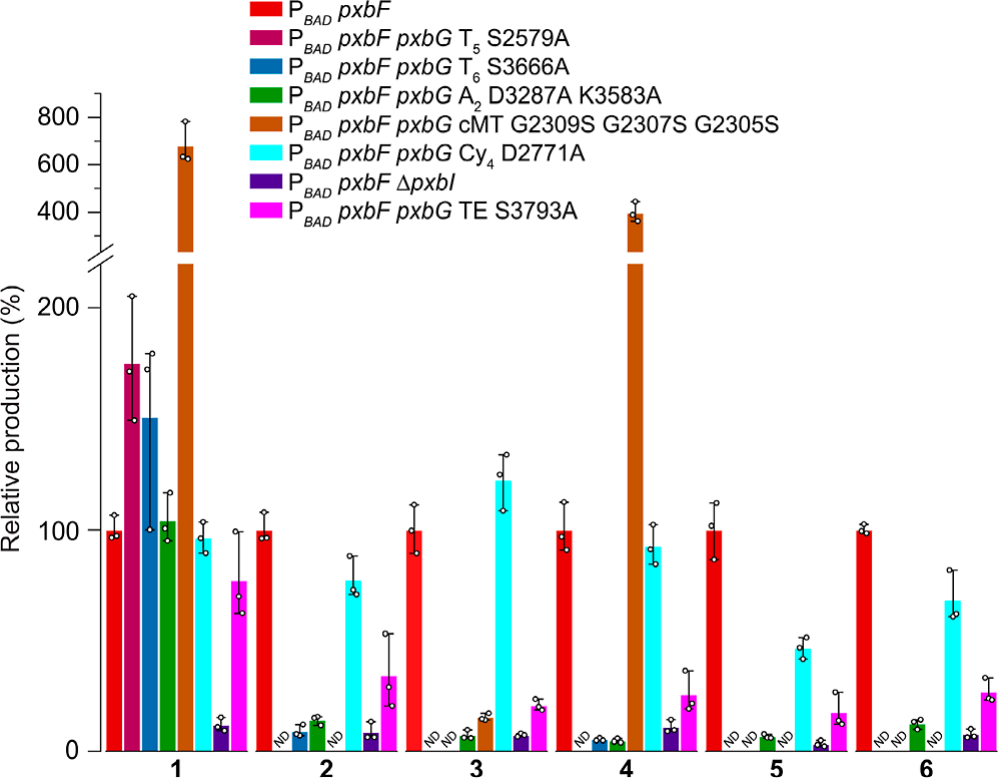
Production of prepiscibactin (**1**), piscibactin (**2**), and photoxenobactins A–D (**3–6**) in different mutants. The production of individual compounds in each inactivation mutant is relative to its production in the *X. szentirmaii* P_*BAD*_*pxbF* mutant (in percentage, %). Data represent mean ± standard deviation from three independent experiments. ND, not detectable.

The observed various chain lengths of piscibactins and photoxenobactins might imply the existence of premature off-loading stages on PxbG, and therefore, we set out to interrupt the assembly line by inactivating T_5_ and T_6_ domains. Inactivation of the T_5_ domain by replacing the serine residue in the conserved motif (LGGDSL) with an alanine residue (PxbG T_5_ S2579A, Figure S2) abolished production of **2**–**6** (Figure 3a traces iv), while led to a 1.6-fold increase in production of **1** (Figure 4), indicating the off-loading of **1** from the T_4_ domain (Figure 2). Inactivation of the T_6_ domain (PxbG T_6_ S3666A, Figure S2) led to accumulation of **1** and complete loss of **5** and **6**, suggesting that **5** and **6** are derived from the T_6_ domain (Figures 2 and 3a trace v). Compounds **2** and **4** absent in the T_5_ inactivation mutant were detected in the T_6_ inactivation mutant but with remarkably reduced production titers (Figure 4), which suggests that they are released from the T_5_ domain (Figure 2). Compound **3** possessing the same chain length as **4** is supposed to be released from the T_5_ domain (this is confirmed by the PxbG cMT inactivation mutant below). However, **3** was barely observed in the T_6_ inactivation mutant (Figure 4), suggesting that **3** is heavily dependent on the product(s) released from the T_6_ domain.

### Putative Cleavage Off-Loading of Photoxenobactins from the Assembly Line

It is curious that although **2**–**4** are released from the T_5_ domain, **3** and **4** are terminated by a thiocarboxylic acid and carboxamide, respectively, instead of a carboxylic acid as in **2**. Also, **5** and **6** derived from the T_6_ domain feature a dithioperoxoate and carboxamide, respectively. At first sight, the termini of photoxenobactins appeared to form through aminolytic and thiolytic releases by nucleophilic attacks of free ammonia and sulfide, which seemed consistent with the hydrolytic release of **2**. However, if this were the case, it would have been observed that those with identical chain lengths (**1** vs **3** and **4** and **2** vs **5** and **6**) would be released from the same T domain. Therefore, off-loadings of photoxenobactins might be achieved in a different manner.

**Figure 5:**
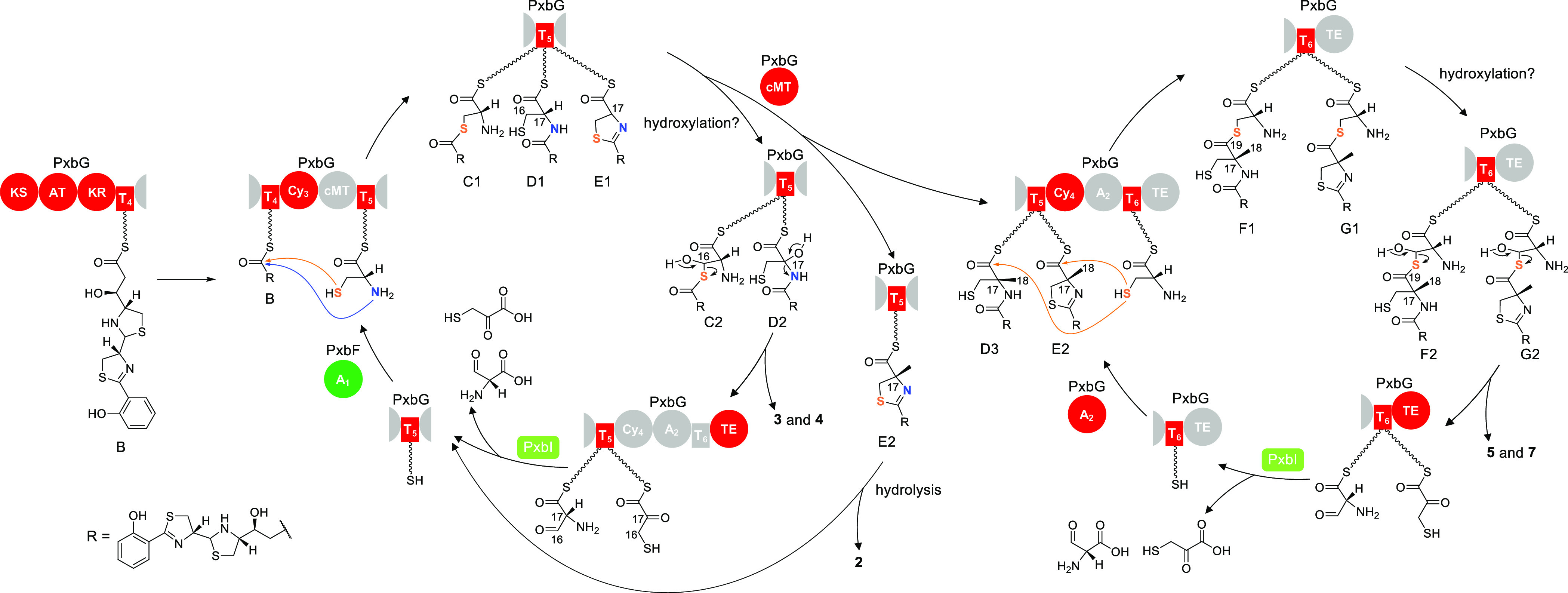
Proposed model for piscibactin and photoxenobactin biosynthesis and the catalytic cycle of the PKS/NRPS assembly line. The domain involved in the corresponding reaction step is highlighted. The amide bond in the intermediate D1 could be formed by the α-amino group of cysteinyl–S–T_5_ directly nucleophilically attacking the upstream acyl–S–T_4_ (intermediate B) under the catalysis of PxbG Cy_3_. Alternatively, PxbG Cy_3_ catalyzes the β-sulfhydryl group of cysteinyl–S–T_5_ forming a thioester with intermediate B, followed by a rapid intramolecular S- to N-acyl transfer, resulting in the amide bond formation. Atypical TE domains have been increasingly identified, exemplified by TE domains catalyzing transesterification,^13,14^ Claisen condensation,^15^ and multiple reactions,^16^ and therefore, we tentatively assigned PxbG TE the role of off-loading the T domain-bound residue of cleavage products, which was indicated by the remarkable decrease in production titers of **3**–**5** in the PxbG TE S3793A mutant.

Non-canonical formation of a terminal carboxamide (e.g., **4**) in biosynthetic assembly lines is reminiscent of the biosynthesis of myxothiazol A. The off-loading of myxothiazol A is mediated by a peptidylglycine α-hydroxylating monooxygenase domain that is encoded in the assembly line and cleaves the glycine residue to afford a terminal carboxamide.^11^ Although no monooxygenase domains are present in the *pxb* BGC, it is tempting to hypothesize that (1) the T_5_ domain is loaded with an l-cysteine by the PxbF A_1_ and/or PxbG A_2_ domain, (2) then the Cy_3_ domain condenses intermediate B–S–T_4_ with cysteinyl–S–T_5_ via amide bond formation to afford the T_5_-bound intermediate D1, in which the amide bond formation is catalyzed by a Cy domain that has been described in in vitro reconstruction of the yersiniabactin biosynthesis,^8,12^ (3) and finally, C-17 methine (the α-carbon of cysteinyl) is hydroxylated (intermediate D2) to trigger spontaneous C–N bond cleavage release of **4** (Figure 5).

To test the hypothesis, we constructed the PxbG A_2_ D3287A K3583A inactivation mutant (Figure S2) that maintained production of **1** but gave rise to lower titers of **2**–**6** (Figures 3a trace vi and 4). This result suggests that the PxbF A_1_ domain recognizing T_2_, T_3_, T_5_, and T_6_ domains in *trans* can partially supplement lost PxbG A_2_ activity, while the PxbG A_2_ domain is more specific for T_5_ and T_6_ domains. Then, we fed the culture of the *X. szentirmaii* P_*BAD*_*pxbF* mutant with l-[U–^13^C,^15^N]-cysteine, in which **4** showed a mass shift of +9 Da (Figure 3b). This is made up of full preservations of two labeled cysteine residues (+8 Da) and an additional ^15^N atom (+1 Da), suggesting that the terminal nitrogen is derived from the α-amino group of cysteine and thus supporting our proposed oxidative cleavage off-loading (Figure 5). On the other hand, given that the PxbG cMT domain (homologous to Ybt cMT_2_, a C-17 methyltransferase domain)^8^ is responsible for introducing a methyl group at C-17 as observed in **2**, **5**, and **6**, we propose that the methylation and the putative hydroxylation have direct competition at C-17 of intermediate D1–S–T_5_. The cMT inactivation mutant (PxbG cMT G2309S G2307S G2305S, Figure S2) resulted in loss of **2**, **5**, and **6** without concomitant formation of their non-methylated products and led to production of **1**, **3**, and **4**, with seven- and four-fold production increase of **1** and **4**, respectively (Figures 3a trace ix and 4). The cMT domain inactivation resulting in remarkable accumulations of **1** and **4** could be explained by the downstream consumption of putative intermediates B and D1 being blocked. These findings not only support the putative C-17 hydroxylation cleavage that leads to the formation of **4** but also indicate that the C-17 methylation step as one of the key checkpoints is a prerequisite for recognition by module 6 and thus suggests that **3** is off-loaded from the T_5_ domain (Figures 2 and 5).

Our previous study showed that four ^34^S atoms were incorporated into **5** by l-[U–^34^S]-cysteine feeding,^1^ suggesting that four cysteines are utilized by modules 2, 3, 5, and 6 to furnish heterocyclic rings and dithioperoxoate. To further demonstrate the sulfur of thioester in the dithioperoxoate moiety derived from the incorporation of a cysteine catalyzed by the Cy_4_ domain, we mutated the first aspartic acid residue in the C3 core motif (DXXXXDXXS) with an alanine residue (PxbG Cy_4_ D2771A, Figure S2). Consistent with previous observation of mutagenesis in the heterocyclization domain on the identical aspartic acid residue,^17^ the PxbG Cy_4_ D2771A mutant compromised the production with a twofold decrease in **5** (Figure 4).

Given that C–S bond cleavages found during the biosyntheses of gliotoxin,^18^ leinamycin,^19^ and lincomycin^20^ are catalyzed by cysteine β-lyases, we, therefore, propose that the sulfur-containing termini of **3**, **5**, and **7** could be formed in a similar manner. Alternatively, a hypothesis where hydroxylation at the β-carbon of cysteinyl (intermediates C2, F2, and G2) triggers a cleavage off-loading cannot be excluded (Figure 5). It is highly likely that **5** is an oxidative form of a cleavage product (intermediate F3) that is prone to form a disulfide bridge via spontaneous oxidation (Figure 2).

### Unusual Termini Might Arise from Low Catalytic Efficiency of Heterocyclization Domains

The formation of a thiazoline unit catalyzed by Cy domains is considered to be a two-step reaction.^2,21^ This involves amide bond formation via the α-amino group of cysteinyl–S–T_*n*_ nucleophilically attacking the upstream acyl/peptidyl–S–T_*n*–1_, identical to the general peptide bond formation catalyzed by an ^L^C_L_, ^D^C_L_, or dual C domain in NRPS biochemistry;^2^ then, cyclodehydration through an attack of the β-sulfhydryl group at carbonyl carbon of the newly formed peptide bond, followed by dehydration of the hemiaminal. Alternatively, the β-sulfhydryl group of cysteinyl–S–T_*n*_ might first form a thioester with the upstream acyl/peptidyl–S–T_*n*–1_, followed by a rapid intramolecular S- to N-acyl transfer, similar to the process of native chemical ligation^22^ used in peptide and protein synthesis and to the recently described mechanism observed during the biosynthesis of clostridial autoinducing peptides.^23^ Therefore, with respect to the terminal thiocarboxylic formation, we surmise that (1) the PxbG Cy domains catalyzing thioesterification is the first step in heterocyclization as the energy barrier for conversion of thioester to amide bonds is much lower than that of amide to thioester bonds^24^ and (2) due to the catalytic inefficiency of the PxbG Cy_3_ and Cy_4_ domains in heterocyclization relative to condensation, the thioester intermediates C1, F1, and G1 might be kinetically stable without being heterocyclized (Figure 5). In particular, the Cy_4_ domain is supposed to be the least efficient due to it only catalyzing thioester formation, albeit sharing ∼60% sequence similarities with Pxb Cy_1–3_ (Figure S3). The finding of thiocarboxylic products (**3**, **5**, and **7**) might serve as a snapshot of the existence of thioester intermediates during thiazoline formation on the *pxb* assembly line. This also indicates that the formation of a thiazoline catalyzed by Cy domains could be a three-step reaction involving a thioester linkage, followed by an S- to N-acyl transfer and subsequent cyclodehydration.

### TE and Type II TE Domains Relevant to Production Titers

Next, we attempted to investigate which enzyme(s) encoded by the *pxb* BGC mediates the cleavage off-loading of photoxenobactins. While the releases of **1** and **2** can be ascribed to spontaneous cyclization and hydrolysis,^7^ respectively (Figure 2), we focused on *pxbI* that encodes a type II thioesterase (TE) homologous to YbtT^25^ and PxbG TE domains for their possible roles in off-loading **3**–**7**. The deletion mutant of *pxbI* only led to a decrease in production titers of all compounds (Figures 3a trace viii and 4), consistent with an earlier report.^26^ The same result was also observed in the PxbG TE inactivation mutant (PxbG TE S3793A, Figures 3a trace vii, 4, and S2). Thus, we assumed that in addition to removing misprimed intermediates and nonreactive acyl residues from T domains,^26^ PxbI might be involved in off-loading the T domain-bound residue of cleavage products, and PxbG TE appears to have an equivalent function (Figure 5). However, we could not rule out the possibility that the acyl/peptidyl intermediates B, C1, D1, E2, F1, and G1 were transferred to the PxbG S3793 residue, where lactamation, hydrolysis, and oxidative cleavage off-loading may then occur. Furthermore, the heterologous expression of a *pxb* homologous BGC from *Photorhabdus luminescens* subsp. *laumondii* TT01 in a non-*pxb*-expressing chassis, *Xenorhabdus doucetiae* FRM16, by CRAGE^27^ also yielded **1**, **2**, **4**, and **5** (Figure S4). Taking all these data into consideration, we proposed that candidate enzyme(s) for mediating photoxenobactin off-loadings might be α- and β-hydroxylases from primary metabolic pathways, which are not encoded by the *pxb* BGC. Our work of seeking the enzyme(s) is still ongoing.

### Post-assembly-Line Conversions of Photoxenobactin C to Other *pxb*-Derived Products

**Figure 6:**
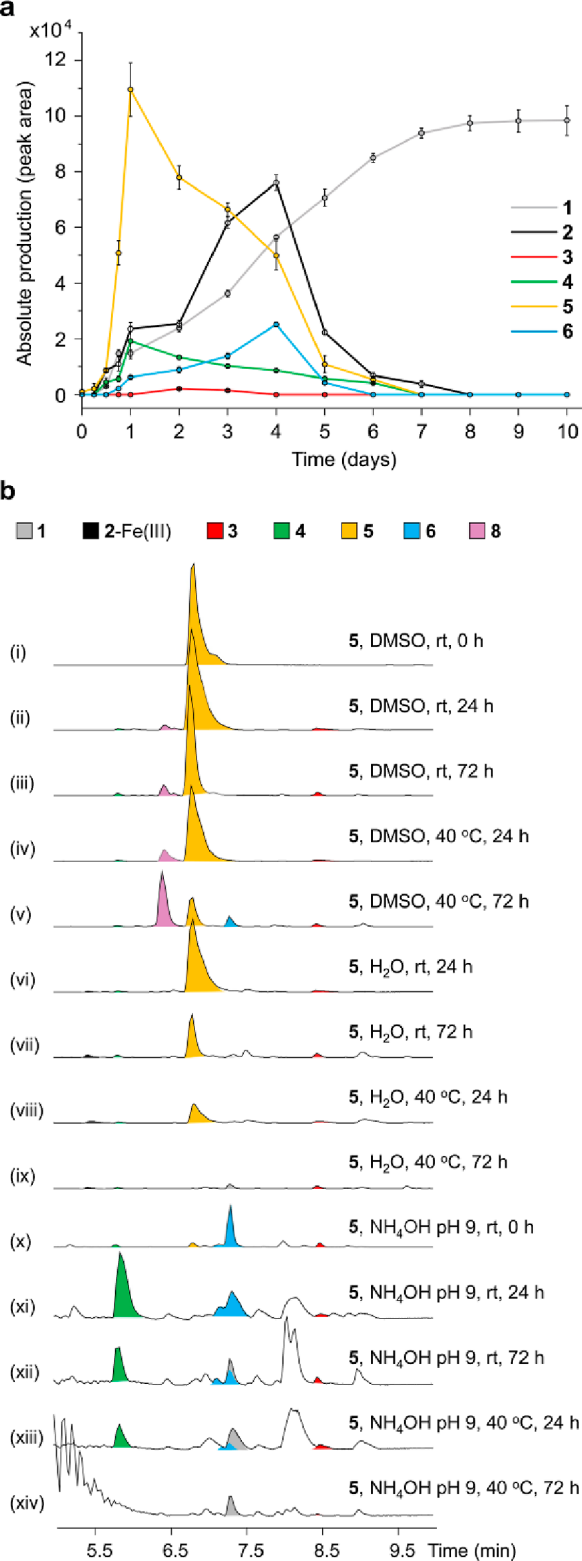
Time course analysis for production of prepiscibactin (**1**), piscibactin (**2**), and photoxenobactins A–D (**3**–**6**) in the culture medium and conversion of photoxenobactin C (**5**) under different conditions. (a) Absolute production of individual compounds in *X. szentirmaii* P_*BAD*_*pxbF* at each time point during a 10 day time course. Data represent mean ± standard deviation from three independent experiments. (b) Incubation of photoxenobactin C (**5**) in DMSO with H_2_O or NH_4_OH (pH 9) at different temperatures. Shown are the BPCs of piscibactins and photoxenobactins.

Interestingly, upon feeding the *X. szentirmaii* P_*BAD*_*pxbF* mutant with l-[U–^13^C,^15^N]-cysteine, we observed that **6** showed a mass shift of +12 Da, which is made up of full preservations of three labeled cysteine residues (Figure 3b), suggesting that the nitrogen in terminal carboxamide is derived from other sources rather than cysteine. Thus, due to **5** and **6** allied to the T_6_ domain as shown above (Figures 2, 3a trace v, and 4), we envisioned **6** as more likely to be a post-assembly-line rearranged product of **5** under basic conditions. Given that **5** with a dithioperoxoate moiety is highly reactive, we speculated that production titers of **2**–**4** are also related to the conversion of **5**, as observed in the dramatic decrease of T_5_ domain-derived products (**2**–**4**) in the T_6_ inactivation mutant (Figures 3a trace v and 4). Then, we carried out a time course analysis of *pxb* production in the *X. szentirmaii* P_*BAD*_*pxbF* mutant (Figure 6a). In contrast to the low-level production of **1**–**4** and **6**, compound **5** was the major component on days 1 and 2. Along with the remarkable reduction of **5**, most of the other compounds’ titers (**1**–**3** and **6**) have various degrees of increases on days 3 and 4. It is conceivable that production of most compounds (**1**–**3** and **6**) in the culture medium is in part derived from the post-assembly-line conversion of **5**. Moreover, the amount of **2**–**6** gradually decreased from day 4 until all disappeared on day 8, while **1** steadily increased, being the only *pxb* product surviving during the whole course. To validate the hypothesis that **5** undergoes spontaneous transformations, we incubated **5** in various solvents and temperatures and monitored the conversion by HPLC–MS (Figure 6b). We observed that in dimethyl sulfoxide (DMSO), a small amount of **5** was converted into **3** and **4** at room temperature and 40 °C in 24 h (Figure 6b traces ii–iv). After 72 h, most of **5** was transformed into photoxenobactin 436 (**8**; Table S1 and Figure S6), which is predicted to be a lactamized product at 40 °C and does not exist in culture media, concomitant with the appearance of **6** (Figure 6b trace v). Incubation of **5** in DMSO with water yielded **2** that formed a complex with iron, as well as **3** and **4** (Figure 6b traces vi–viii). Such an incubation at 40 °C for 72 h gave rise to complete degradation of **5**, along with the formation of **1** (Figure 6b trace ix). To simulate the basic environment of the culture medium, we incubated **5** in DMSO with ammonium hydroxide at pH 9. As expected, under this condition we observed a rapid conversion of **5** into **3**, **4**, **6**, and others, among which **4** and **6** with a terminal carboxamide were the major products (Figure 6b traces x and xi). Upon longer incubation or heating (Figure 6b traces xii–xiv), **1** was formed, and particularly, it was the major *pxb* product that existed in bases at 40 °C after 3 days (Figure 6b trace xiii). Taking all these data into consideration, we reasoned that **5** is a thermodynamically unstable biosynthetic end product and spontaneously converts into other *pxb* products (Figure S7), while **1** is the major thermodynamically stable product resulting from prolonged cultivation.

In summary, we unravel a previously unknown PKS/NRPS biosynthetic logic that utilizes heterocyclization domains with distinct efficiencies to extend a T domain-tethered acyl/peptidyl with a cysteinyl. This leads to the formation of thiazoline which is a mature heterocyclic product, demonstrating a fully functional Cy domain and the formation of amide or thioester, which is a nascent condensation product only accounting for a partly or inadequately functional Cy domain. The resulting T domain-bound acyl/peptidyl on PxbG is off-loaded by intramolecular cyclization, hydrolysis, and putative C–N/C–S bond cleavage, which gives rise to products with different chain lengths featuring carboxylic acid, carboxamide, thiocarboxylic acid, and dithioperoxoate termini. It turned out that photoxenobactin C (**5**) with a dithioperoxoate terminus that is the final biosynthetic product is prone to conversion into the other *pxb* products (**1**–**4** and **6**). Such post-assembly-line spontaneous conversions (Figures 2 and S7) contribute to major titers of **1**–**4** and **6** in the culture medium. Indeed, homologous *pxb* BGCs are not only prevalent across *Xenorhabdus* and *Photorhabdus* but also widespread in other γ-Proteobacteria, such as *Psychromonas*, *Salinivibrio*, *Shewanella*, and *Vibrio* (Figure S5), which offers opportunities to study the BGC evolution and underlying structural diversity that might result from the catalytic discrepancy of Cy domain(s) and enzymes mediating bond cleavage off-loadings. Again, the instability of most *pxb* products might explain why these compounds had not been described from other strains before. Our discoveries thus expand the functional diversity of natural product biosynthetic assembly lines and enhance our understanding of nature’s potential capability to achieve chemical innovation.

## Supporting Information Available

Complete materials and methods, HPLC–MS chromatograms, sequence alignments, phylogenetic tree, and proposed mechanisms of post-assembly-line conversions (PDF)
