# Supplementary material for: Cleavage Off-Loading and Post-assembly-Line Conversions Yield Products with Unusual Termini during Biosynthesis
Source: ACS Chem Biol. 2022 Jul 21;17(8):2221–8. doi: 10.1021/acschembio.2c00367 (PMC9396620; doi:10.1021/acschembio.2c00367)
Supplement: Supplementary file 1 — cb2c00367_si_001.pdf [file cb2c00367_si_001.pdf]

Supporting Information

Cleavage Off-loading and Post-assembly-line Conversions Yield Products with Unusual Termini during Biosynthesis

Yi-Ming Shi*[,1,2], Merle Hirschmann[1], Yan-Ni Shi[1,2], Helge B. Bode[*,1,2,3,4]

[1]Department of Natural Products in Organismic Interactions, Max Planck Institute for Terrestrial Microbiology, 35043 Marburg, Germany

[2]Molecular Biotechnology, Department of Biosciences, Goethe University Frankfurt, 60438 Frankfurt am Main, Germany

[3]Chemical Biology, Department of Chemistry, Philipps University Marburg, 35043 Marburg, Germany

[4]Senckenberg Gesellschaft für Naturforschung, 60325 Frankfurt am Main, Germany

*Corresponding author e-mail: yi-ming.shi@mpi-marburg.mpg.de; helge.bode@mpi-marburg.mpg.de

Methods

General experimental procedures

All chemicals were purchased from Sigma-Aldrich, Acros Organics, or Iris BIOTECH. Isotope-labeled chemicals were purchased from Cambridge Isotope Laboratories, Inc. Genomic DNA of selected *Xenorhabdus* and *Photorhabdus* strains were isolated using the Qiagen Gentra Puregene Yeast/Bact Kit. DNA polymerases (Taq, Phusion, and Q5) and restriction enzymes were purchased from New England Biolabs or Thermo Fisher Scientific. DNA primers were purchased from Eurofins MWG Operon. PCR amplifications were carried out on thermocyclers (SensoQuest). Polymerases were used according to the manufacturers' instructions. DNA purification was performed from 1% TAE agarose gel using Invisorb® Spin DNA Extraction Kit (STRATEC Biomedical AG). Plasmids in *E. coli* were isolated by alkaline lysis. HPLC–UV–MS analysis was conducted on an UltiMate 3000 system (Thermo Fisher) coupled to an AmaZonX mass spectrometer (Bruker) with an ACQUITY UPLC BEH C18 column (130 Å, 2.1 mm × 100 mm, 1.7 µm particle size, Waters) at a flow of 0.6 mL/min (5–95% acetonitrile/water with 0.1% formic acid, v/v, 16 min, UV detection wavelength 190–800 nm). HPLC–UV–HRMS analysis was conducted on an UltiMate 3000 system (Thermo Fisher) coupled to an Impact II qTof mass spectrometer (Bruker) with an ACQUITY UPLC BEH C18 column (130 Å, 2.1 mm × 100 mm, 1.7 µm particle size, Waters) at a flow of 0.4 mL/min (5–95% acetonitrile/water with 0.1% formic acid, v/v, 16 min, UV detection wavelength 190–800 nm).

Strain and culture conditions

The wild-type strain and the mutants thereof (Table S3) were cultivated on lysogeny broth (LB) agar plates at 30 °C overnight and were subsequently inoculated into liquid LB culture at 30 °C with shaking at 200 rpm. For compound production, the overnight LB culture was transferred into 5 mL Sf-900™ II SFM medium (1:100, v/v) with 2% (v/v) of Amberlite™ XAD-16 resins, 0.1 % of L-arabinose as the inducer, and selective antibiotics such as ampicillin (Am, 100 µg/mL), kanamycin (Km, 50 µg/mL), or chloramphenicol (Cm, 34 µg/mL) at 30 °C with shaking at 200 rpm.

Culture extraction and HPLC–UV–MS analysis

The XAD-16 resins were collected after 72 h and extracted with 5 mL ethyl acetate. The solvent was dried under rotary evaporators, and the dried extract was resuspended in 500 µL acetonitrile/water (1:1, v/v, for photoxenobactins), of which 5 µL was injected and analyzed by HPLC–UV–MS or HPLC–UV–HRMS. Unless otherwise specified, HPLC–UV–MS and HPLC–UV–HRMS chromatograms in the figures are shown on the same scale. Relative quantification was measured by the peak area of extracted ion chromatograms (EICs), and peak areas were normalized by OD_{600} values at the harvesting time point.

Time-course analysis

2 mL overnight *X. szentirmaii* P_{BAD} *pxbF* culture was transferred into 200 mL Sf-900 medium with 0.1 % of L-arabinose and kanamycin (50 µg/mL). Cultivation was carried out at 30 °C with shaking at 200 rpm. 100 µL of the culture was taken from the culture at 0, 6, 12, 18, 24, 48, 72, 96, 120, 144, 168, 192, 216, 240 h and stored at -80 °C. The culture was centrifuged and 5 µL supernatant was subjected for HPLC-UV-HRMS. The peak areas of compounds' EICs were quantified using DataAnalysis.

Construction of deletion mutants and point mutation

A ~1000-bp upstream and a ~1000-bp downstream fragments (mutations were introduced by primers) of a target gene (*pxbH*, *pxbI*, *pxbJ*, *pxbG* T_5, *pxbG* T_6, *pxbG* A_2, *pxbG* cMT, *pxbG* Cy_4, and *pxbG* TE) were amplified using primer pairs listed in Table S5. The amplified fragments were fused using the complementary overhangs introduced by primers and cloned into the pCKcipB or pEB17 vector (linearized with PstI and BglII) by Hot Fusion.[1] Transformation of *E. coli* S17-1 *λ* pir with the resulting plasmid and conjugation with a wild-type strain, as well as the generation of double crossover mutants via counterselection on LB plates containing 6% sucrose. Deletion mutants were verified via PCR using primer pairs listed in Table S5, which yielded a ~2000-bp fragment for mutants genetically equal to the WT strain and a ~1000-bp fragment for the desired deletion mutant. Point mutations were verified by sequencing a ~1000-bp PCR product that carries desired mutation(s).

Heterologous expression of *pxb* BGC

The *pxb* BGC from *P. luminescens* subsp. *laumondii* TT01was integrated into the genome of a non-photoxenobactin-producing strain, *X. doucetiae* FRM16, by CRAGE for heterologous expression as described[2].

Labeling experiments

The cultivation of strains for labeling experiments was carried out as described above. The overnight culture was transferred into LB medium additionally fed with L-[U-^{13}C,^{15}N]cysteine at a final concentration of 1 mM.

Data availability

All data generated or analyzed in this study are available within the article and its Supporting Information file. The genome sequence data involved in this study are accessible in NCBI GenBank database under an accession number NIBV00000000 (*X. szentirmaii* DSM 16338).

Table S1. HR–ESI–MS data of photoxenobactins described in this work.

Compound	Detected mass	Calculated mass	Error (ppm)	Ion formula
prepiscibactin (**1**)	337.0662 $[M + H]^+$	337.0675 $[M + H]^+$	3.9	$C_{15}H_{17}N_2O_3S_2$ $[M + H]^+$
piscibactin (**2**)	454.0910 $[M + H]^+$	454.0924 $[M + H]^+$	3.0	$C_{19}H_{24}N_3O_4S_3$ $[M + H]^+$
photoxenobactin A (**3**)	353.0426 $[M – H_2O + H]^+$	353.0447 $[M – H_2O + H]^+$	5.9	$C_{15}H_{17}N_2O_2S_3$ $[M – H_2O + H]^+$
photoxenobactin B (**4**)	336.0837 $[M – H_2O + H]^+$	336.0835 $[M – H_2O + H]^+$	-0.6	$C_{15}H_{18}N_3O_2S_2$ $[M – H_2O + H]^+$
photoxenobactin C (**5**)	468.0526 $[M – H_2O + H]^+$	468.0539 $[M – H_2O + H]^+$	2.7	$C_{19}H_{22}N_3O_3S_4$ $[M – H_2O + H]^+$
photoxenobactin D (**6**)	453.1065 $[M + H]^+$	453.1083 $[M + H]^+$	4.0	$C_{19}H_{25}N_4O_3S_3$ $[M + H]^+$
photoxenobactin E (**7**)	470.0695 $[M + H]^+$	470.0695 $[M + H]^+$	0.0	$C_{19}H_{24}N_3O_3S_4$ $[M + H]^+$
Photoxenobactin 436 (**8**, predicted)	436.0807 $[M + H]^+$	436.0818 $[M + H]^+$	2.5	$C_{19}H_{22}N_3O_3S_3$ $[M + H]^+$

Table S2. Putative functional assignments of biosynthetic genes in the *pxb* BGC.

Protein	Locus tag	Homolog (accession number, organism)	Identified/Proposed function	Coverage/Identity (%)
PxbA	Xsze_RS15545	YbtA (BBK40901.1, *Yersinia pestis*)	AraC family transcriptional regulator	96/15
PxbB	Xsze_RS15540	Psn (CAJ87595.1, *Yersinia pestis*)	TonB-dependent siderophore receptor	98/29
PxbC	Xsze_RS15535	YbtX (BAM09331.1, *Yersinia pestis*)	MFS family transporter	100/30
PxbD	Xsze_RS15530	YbtQ (CAD15510.1, *Yersinia pestis*)	ABC transporter	96/30
PxbE	Xsze_RS15525	YbtP (CAD15510.1, *Yersinia pestis*)	ABC transporter	100/28
PxbF	Xsze_RS15520	HMWP2 (CAJ87590.1, *Yersinia pestis*)	NRPS	98/47
PxbG	Xsze_RS15515	HMWP1 (CAJ87591.1, *Yersinia pestis*)	PKS/NRPS	79/32
PxbH	Xsze_RS15510	YbtU (CAJ87592.1, *Yersinia pestis*)	reductase	100/42
PxbI	Xsze_RS15505	YbtT (CAJ87593.1, *Yersinia pestis*)	thioesterase	100/45
PxbJ	Xsze_RS15500	YbtE (CAJ87594.1, *Yersinia pestis*)	2,3-dihydroxybenzoate-AMP ligase	53/52
		YbtS (CAJ87585.1, *Yersinia pestis*)	salicylate synthase	44/31

Table S3. Strains used in this study.

Strain	Genotype/Description	Reference
X.szentirmaii DSM 16338	wild type	[3]
X. szentirmaii P_{BAD} *pxbF*	*X. szentirmaii* pCEP *Xsze_RS15520*, *araBAD* promoter, Km^r	[4]
X. szentirmaii P_{BAD} *pxbF* Δ*pxbJ*	*X. szentirmaii* pCEP *Xsze_RS15520*, Δ*Xsze_RS15500*, *araBAD* promoter, Km^r	This study
X. szentirmaii P_{BAD} *pxbF* Δ*pxbJ*::*pxbJ*	pACYC *araBAD Xsze_RS15500* complementation of *X. szentirmaii* pCEP *Xsze_RS15520*, Δ*Xsze_RS15500*, *araBAD* promoter, Km^r, Cm^r	This study
X. szentirmaii P_{BAD} *pxbF* Δ*pxbH*	*X. szentirmaii* pCEP *Xsze_RS15520*, Δ*Xsze_RS15510*, *araBAD* promoter, Km^r	This study
X. szentirmaii P_{BAD} *pxbF* Δ*pxbH*::*pxbH*	pACYC *araBAD Xsze_RS15510* complementation of *X. szentirmaii* pCEP *Xsze_RS15520*, Δ*Xsze_RS15510*, *araBAD* promoter, Km^r, Cm^r	This study
X. szentirmaii P_{BAD} *pxbF* Δ*pxbI*	*X. szentirmaii* pCEP *Xsze_RS15520*, Δ*Xsze_RS15505*, *araBAD* promoter, Km^r	This study
X. szentirmaii P_{BAD} *pxbF pxbG* T_5 S2579A	*X. szentirmaii* pCEP *Xsze_RS15520*, *Xsze_RS15515* S2579A, *araBAD* promoter, Km^r	This study
X. szentirmaii P_{BAD} *pxbF pxbG* T_6 S3666A	*X. szentirmaii* pCEP *Xsze_RS15520*, *Xsze_RS15515* S3666A, *araBAD* promoter, Km^r	This study
X. szentirmaii P_{BAD} *pxbF pxbG* A_2 D3287A K3583A	*X. szentirmaii* pCEP *Xsze_RS15520*, *Xsze_RS15515* D3287A K3583A, *araBAD* promoter, Km^r	This study
X. szentirmaii P_{BAD} *pxbF pxbG* cMT G2309S G2307S G2305S	*X. szentirmaii* pCEP *Xsze_RS15520*, *Xsze_RS15515* G2309GS, G2307GS, G2305S, *araBAD* promoter, Km^r	This study
X. szentirmaii P_{BAD} *pxbF pxbG* TE S3793A	*X. szentirmaii* pCEP *Xsze_RS15520*, *Xsze_RS15515* S3793A, *araBAD* promoter, Km^r	This study
X. szentirmaii P_{BAD} *pxbF pxbG* Cy_4 D2771A	*X. szentirmaii* pCEP *Xsze_RS15520*, *Xsze_RS15515* D2771A, *araBAD* promoter, Km^r	This study
XP05_BGC5	*X. doucetiae* FRM16 with a landing pad at 559513, *PLU_RS11540-11585* integrated into the chromosome, *lox*, *T7* promoter, apramycin^r	[2]

Table S4. Plasmids used in this work.

Plasmid	Genotype/Description	Reference
pCKcipB	pDS132 derivative with an additional *Bgl*II recognition site, R6Kγ *ori*, *oriT*, *cipB* promoter, Cm^r	[5]
pEB17_KM	pDS132 derivative with an additional *Bgl*II recognition site, R6Kγ *ori*, *oriT*, *cipB* promoter, Km^r	[6]
pACYC *araBAD tacI*	pACYCDuet-1, *lacI*::*araC araBAD*	Bode lab collection
pCKcipB *pxbJ*	pCKcipB *Xsze_RS15500'*, *cipB* promoter, Cm^r	This study
pACYC *pxbJ*	pACYC *araBAD tacI Xsze_RS15500*, *araBAD* promoter, Cm^r	This study
pCKcipB *pxbH*	pCKcipB *Xsze_RS15510'*, *cipB* promoter, Cm^r	This study
pACYC *pxbH*	pACYC *araBAD tacI Xsze_RS15510*, *araBAD* promoter, Cm^r	This study
pCKcipB *pxbI*	pCKcipB *Xsze_RS15505'*, *cipB* promoter, Cm^r	This study
pCKcipB *pxbG* T_5 S2579A	pCKcipB *Xsze_RS15515', Xsze_RS15515* S2579A, *cipB* promoter, Cm^r	This study
pCKcipB *pxbG* T_6 S3666A	pCKcipB *Xsze_RS15515', Xsze_RS15515* S3666A, *cipB* promoter, Cm^r	This study
pEB17_KM *pxbG* A_2 D3287A	pCKcipB *Xsze_RS15515', Xsze_RS15515* D3287A, *cipB* promoter, Km^r	This study
pCKcipB *pxbG* A_2 K3583A	pCKcipB *Xsze_RS15515', Xsze_RS15515* K3583A, *cipB* promoter, Cm^r	This study
pCKcipB *pxbG* cMT G2309S G2307S G2305S	pCKcipB *Xsze_RS15515', Xsze_RS15515* G2309S G2307S G2305S, *cipB* promoter, Cm^r	This study
pCKcipB *pxbG* TE S3793A	pCKcipB *Xsze_RS15515', Xsze_RS15515* S3793A, *cipB* promoter, Cm^r	This study
pCKcipB *pxbG* Cy_4 D2771A	pCKcipB *Xsze_RS15515', Xsze_RS15515* D2771A, *cipB* promoter, Cm^r	This study

Table S5. Primers used in this work.

Primer	Sequence (5'-3')	Purpose
pCEP-Ve-Fw	GCTATGCCATAGCATTTTTATCCATAAG	Verification of the pCEP_kan and pCEP_cm constructs
pDS132-Ve-Rv	ACATGTGGAATTGTGAGCGG	
pCOLA_araP_tacI_Gib_fw	CTGCAGGAGCTGTTGACAAT	Backbone amplification of pACYC *araBAD tacI*
pCOLA_araP_tacI_Gib_rv	CATGGAATTCCTCCTGTTAGC	
MHp139	TCTAGAGTCGACCTGCAGAGAATGTGACGGTGCTGAAGG	Deletion of *pxbJ*
MHp140	GTTTAATGCTGTCATTCGCGTTATGCGAACTCCTGTGGA	
MHp141	TCCACAGGAGTTCGCATAACGCGAATGACAGCATTAAAC	
MHp142	CCGGGAGAGCTCAGATCTGGTGTTGCCTGACAACCTATG	
MHp143	CTAGAGTCGACCTGCAGACGTCTGATGATGAAAATCAAC	Deletion of *pxbH*
MHp144	CAGGGAGTGTCCATTTCATTCATATGTTCTCCTTAACAGG	
MHp145	GTTAAGGAGAACATATGAATGAAATGGACACTCCCTG	
MHp146	CGGGAGAGCTCAGATCTGGATCCTGATAGATCAGTGCCG	
YS-Pxb-D1-Fw	TCGATCCTCTAGAGTCGACCTGCAGACGTTCATTGGATGAATTG	Deletion of *pxbI*
YS-Pxb-D1-Rv	GGGATTGAATTAAGTGCCATTCAGCATTCTTCCTTGGC	
YS-Pxb-D2-Fw	TGGCCAAGGAAGAATGCTGAATGGCACTTAATTCAATCCC	
YS-Pxb-D2-Rv	AATTCCCGGGAGAGCTCAGATCTGACCTGTTGCAGAGGGCC	
MHp176	CTAACAGGAGGAATTCCATGGCACTTAATTCAATCCCGG	Complementation of *pxbJ*
MHp177	ATTGTCAACAGCTCCTGCAGTTAGGAATGGACATATTTCATA	
MHp178	CTAACAGGAGGAATTCCATGATAAAGCCACAACGGGTT	Complementation of *pxbH*
MHp179	ATTGTCAACAGCTCCTGCAGTCAGCATTCTTCCTTGGCC	
YS-Pxb-T5-Fw1	CCTCTAGAGTCGACCTGCAGCGACCTGTATCGAACAACATGAC	Point mutation of *pxbG* T_5 S2579A
YS-Pxb-T5-Rv1	GACCATGCGGGTCGCAATCAATGCATCCCCGCCACTTTGGAAA	
YS-Pxb-T5-Fw2	CCAAAGTGGCGGGGATGCATTGATTGCGACCCGCATGG	
YS-Pxb-T5-Rv2	TCCCGGGAGAGCTCAGATCTTTGCCATGCAAGACATTGATCG	
MHp212	ATCCTCTAGAGTCGACCTGCAGAGGTAACCGGTGAGCTGTAT	Point mutation of *pxbG* T_6 S3666A
MHp213	GCACCGCGGCAAGTGCATCTCCGCCAAGAACAAAG	
MHp214	TTCTTGGCGGAGATGCACTTGCCGCGGTGCGTCT	
MHp215	TCCCGGGAGAGCTCAGATCTTAATCGACCAATGCGCGC	
YS-Pxb-A2-Fw1	CCTCTAGAGTCGACCTGCAGACGTGTTATCTCTCTGGATGAAAC	Point mutation of *pxbG* A_2 K3583A
YS-Pxb-A2-Rv1	CATTAACGTCCGTCTGTCGATTGCACCGTTGTCGGAAACAGGC	
YS-Pxb-A2-Fw2	GCCTGTTTCCGACAACGGTGCAATCGACAGACGGACGTTAATG	
YS-Pxb-A2-Rv2	TCCCGGGAGAGCTCAGATCTGTCGAGATAATCCATAAAATCAGGC	
YS-Pxb-A2-Fw3	CCTCTAGAGTCGACCTGCAGCAATGGCTGGCTGATCTTCAAC	Point mutation of *pxbG* A_2 D3287A
YS-Pxb-A2-Rv3	GAATAAGTCGTAGACGGAGAGTGCGAAAGTTAATTCTGAGATTGCCAG	
YS-Pxb-A2-Fw4	GGCAATCTCAGAATTAACTTTCGCACTCTCCGTCTACGACTTATTC	
YS-Pxb-A2-Rv4	TCCCGGGAGAGCTCAGATCTTCCAGATTGGCAGAGTTTGCTG	
YS-Pxb-MT-Fw1	CCTCTAGAGTCGACCTGCAGGCAAGAAGGCGTGGCTATC	Point mutation of *pxbG* cMT G2309S G2307S G2305S
YS-Pxb-MT-Rv1	GTGATGTAGCCGATGTACTTGCTGAGACTTCCAGCATCGTGAAATGC	
YS-Pxb-MT-Fw2	TGCTGGAAGTCTCAGCAAGTACATCGGCTACATCACAAAAAGTGCTTAAAC	
YS-Pxb-MT-Rv2	TCCCGGGAGAGCTCAGATCTGAATTTCTTCCGATGCCAAAGC	
MHp202	CGATCCTCTAGAGTCGACCTGCAGACTCTGCCAATCTGGATGTGC	Point mutation of *pxbG* TE S3793A
MHp203	AATCCGCCAAATGCCCAGCCTGCCAGTGCAT	
MHp204	CAGGCTGGGCATTTGGCGGATTAGTCGCCTA	
MHp205	GAATTCCCGGGAGAGCTCAGATCTATCACGGGCAAGCTGCTGT	
YS-Pxb-Cy4-Fw1	CCTCTAGAGTCGACCTGCAGAATCATGATGATAATGAGAGTGC	Point mutation of *pxbG* Cy_4 D2771A
YS-Pxb-Cy4-Rv1	CTTTTTCCGTCCGCGACCACTAACGCAACGGTCAAATGCAATAGATATG	
YS-Pxb-Cy4-Fw2	CATATCTATTGCATTTGACCGTTGCGTTAGTGGTCGCGGACGGAAAAAG	
YS-Pxb-Cy4-Rv2	TCCCGGGAGAGCTCAGATCTCGGCAGTGCGTTGTGGATAAC	

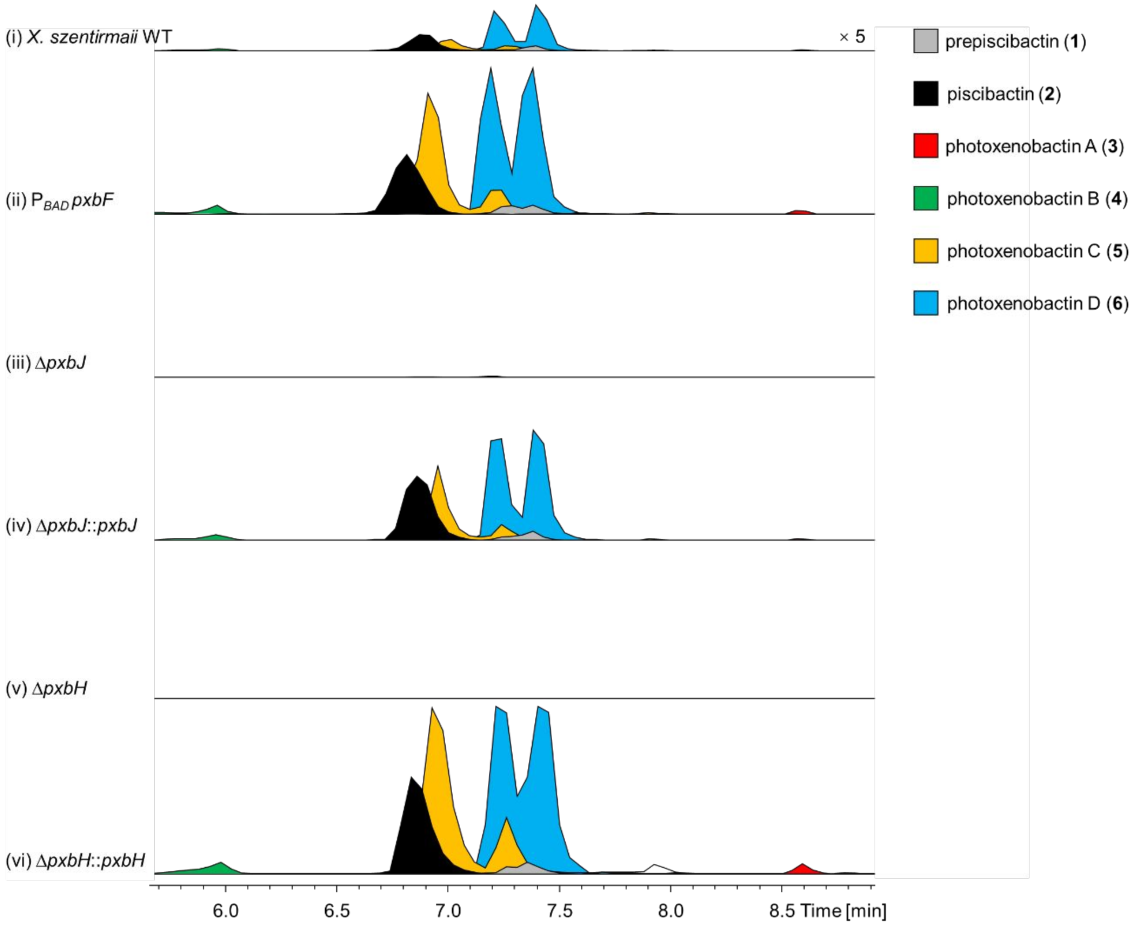

Figure S1. HPLC–MS analysis of the wild-type strain, as well as deletion and complementation mutants in Sf-900 medium. Mutants were induced with L-arabinose. Shown are the EICs of prepiscibactin (**1**, grey), piscibactin (**2**, black), and photoxenobactins A (**3**, red), B (**4**, green), C (**5**, yellow), and D (**6**, blue). Each compound contains a pair of C-10 epimers which is resulted from the thiazolinyl–S–PCP_2 reduction by PxbH. All epimers were not differentiated. Intensities of EICs in (i) are magnified for visualizing tiny peaks. Magnifications are indicated on the right side of traces.

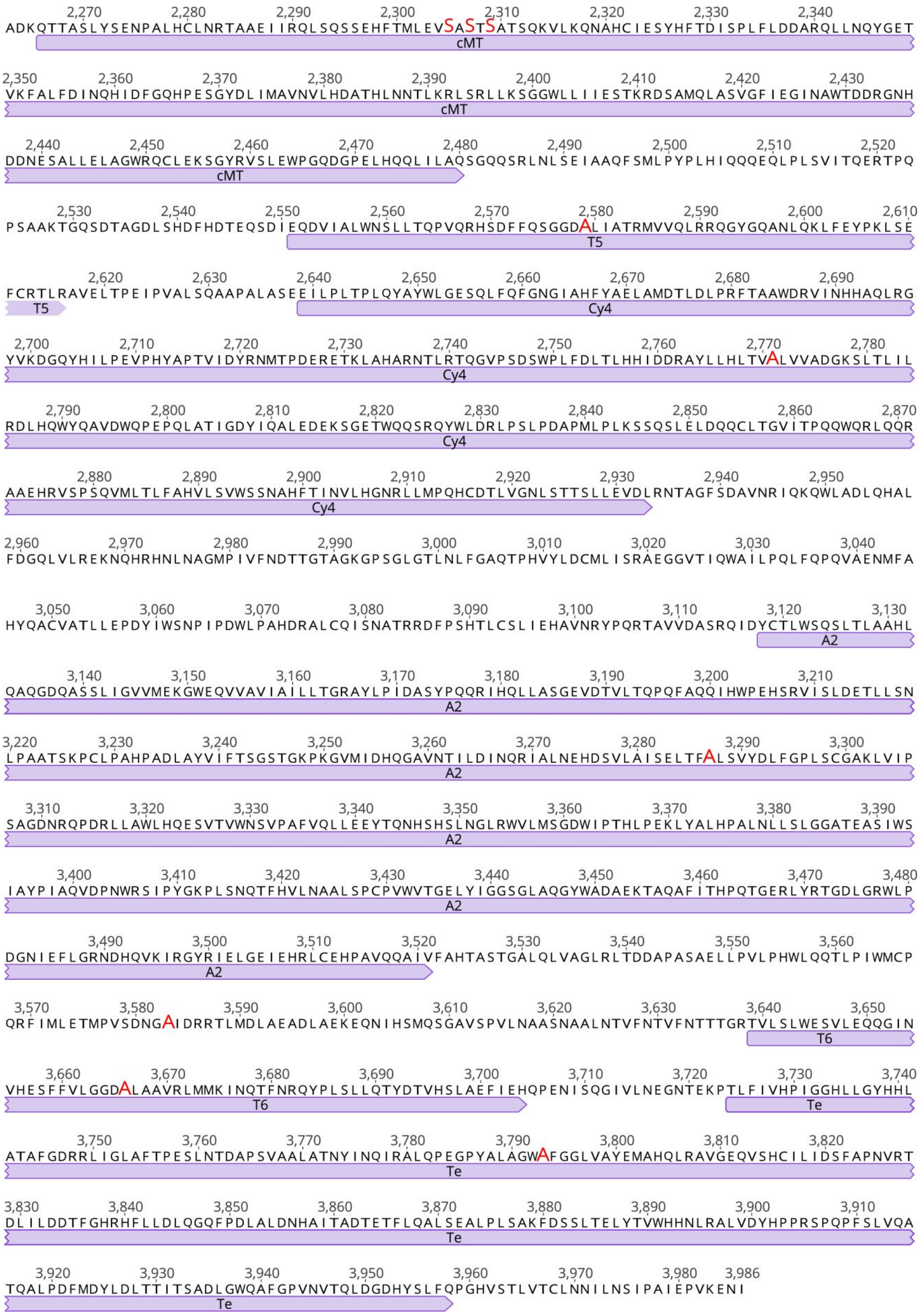

Figure S2. Point mutations of PxbG. Mutated residues are colored red.

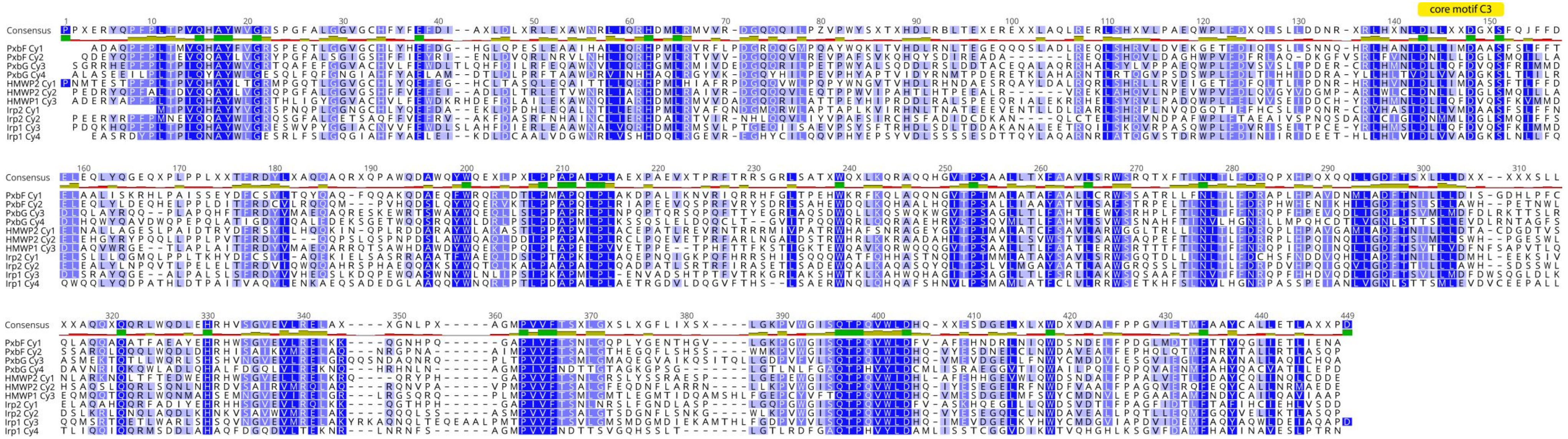

	Irp1 Cy3	Irp1 Cy4	Irp2 Cy1	Irp2 Cy2	PxbF Cy1	PxbF Cy2	PxbG Cy3	PxbG Cy4	HMWP1 Cy3	HMWP2 Cy1	HMWP2 Cy2
Irp1 Cy3		61%	62%	65%	64%	65%	77%	61%	78%	61%	66%
Irp1 Cy4	61%		61%	63%	61%	62%	61%	73%	63%	58%	60%
Irp2 Cy1	62%	61%		66%	78%	68%	66%	60%	66%	75%	65%
Irp2 Cy2	65%	63%	66%		63%	80%	65%	59%	66%	65%	76%
PxbF Cy1	64%	61%	78%	63%		67%	66%	60%	65%	79%	64%
PxbF Cy2	65%	62%	68%	80%	67%		67%	61%	69%	65%	80%
PxbG Cy3	77%	61%	66%	65%	66%	67%		63%	80%	63%	66%
PxbG Cy4	61%	73%	60%	59%	60%	61%	63%		64%	57%	60%
HMWP1 Cy3	78%	63%	66%	66%	65%	69%	80%	64%		62%	68%
HMWP2 Cy1	61%	58%	75%	65%	79%	65%	63%	57%	62%		65%
HMWP2 Cy2	66%	60%	65%	76%	64%	80%	66%	60%	68%	65%	

Figure S3. Multiple sequence alignment of Cy domains from *pxb* (PxbF $Cy_{1,2}$ and PxbG $Cy_{3,4}$), *ybt* (HMWP2 $Cy_{1,2}$ and HMWP1 Cy_3), and *irp* (Irp $Cy_{1,2}$ and Irp $Cy_{3,4}$) BGCs. The core motif C3 contains signature DXXXXDXXS. Protein sequence similarity analysis of Cy domains using Geneious Prime Blosum45 (threshold = 0) scoring matrix.

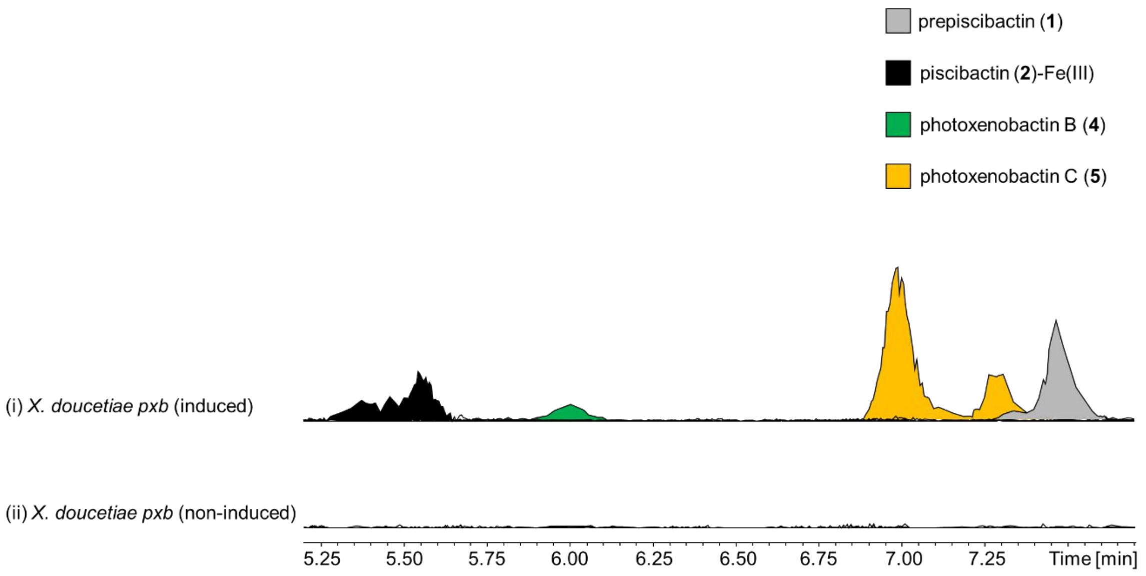

Figure S4. HPLC–MS analysis of *X. doucetiae* FRM16, a non-photoxenobactin-producing strain, expressing the *pxb* BGC from *P. luminescens* TT01 by CRAGE[2] in XPP medium with/without IPTG induction. Shown are the EICs of prepiscibactin (**1**, grey), piscibactin (**2**)-Fe^{III} (black), and photoxenobactins B (**4**, green) and C (**5**, yellow).

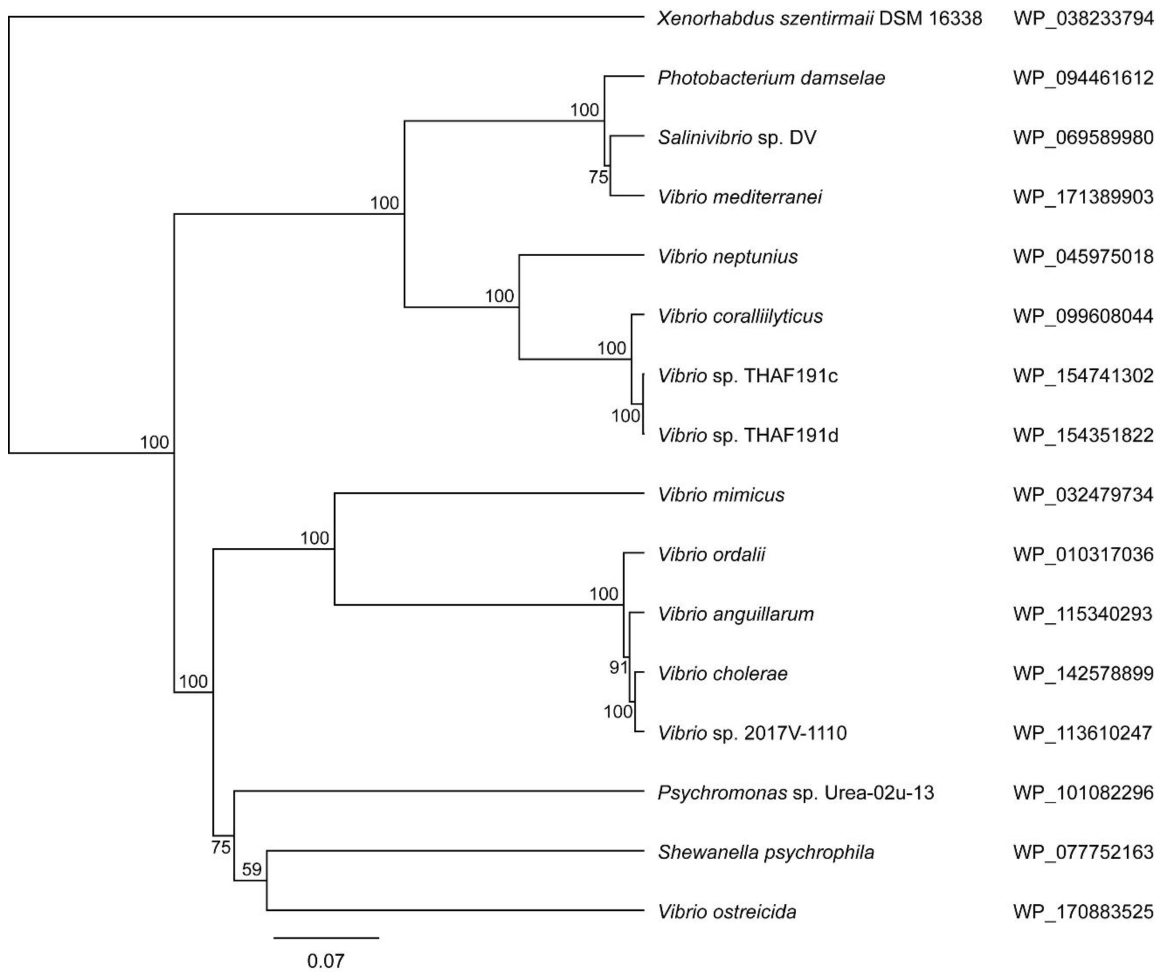

Figure S5. Phylogenetic tree (UPGMA) based on the PKS/NRPS protein sequence of homologous *pxb*/*irp* BGCs. Protein sequences of PxbG homologs were obtained from the BLAST search of PxbG/Irp1 to the NCBI database. The numbers next to the branches represent the percentage of consensus support in which this topology was reached in a bootstrap test of 100 replicates. Accession numbers of each protein are indicated on the right side.

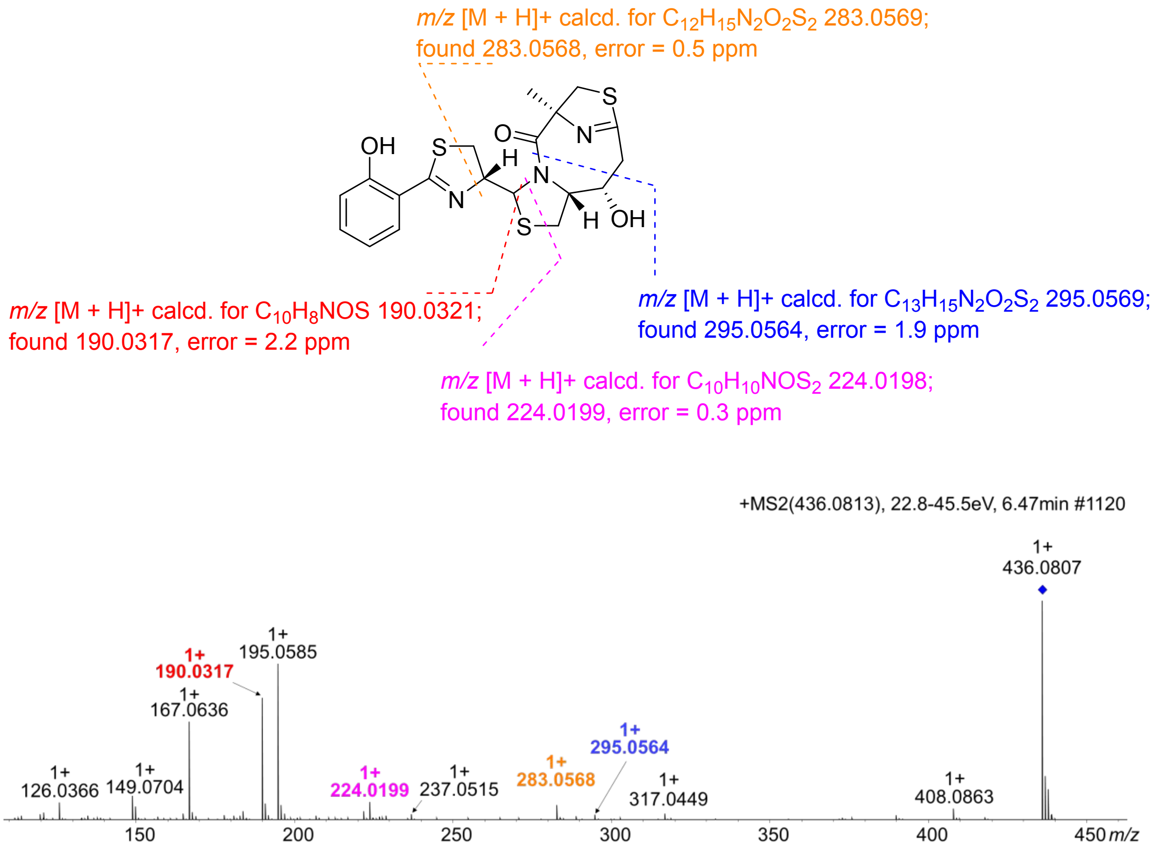

Figure S6. MS/MS fragmentation patterns of photoxenobactin 437 (**8**). The blue diamond indicated the parent ion. The MS fragments that help with predicting the structure are highlighted.

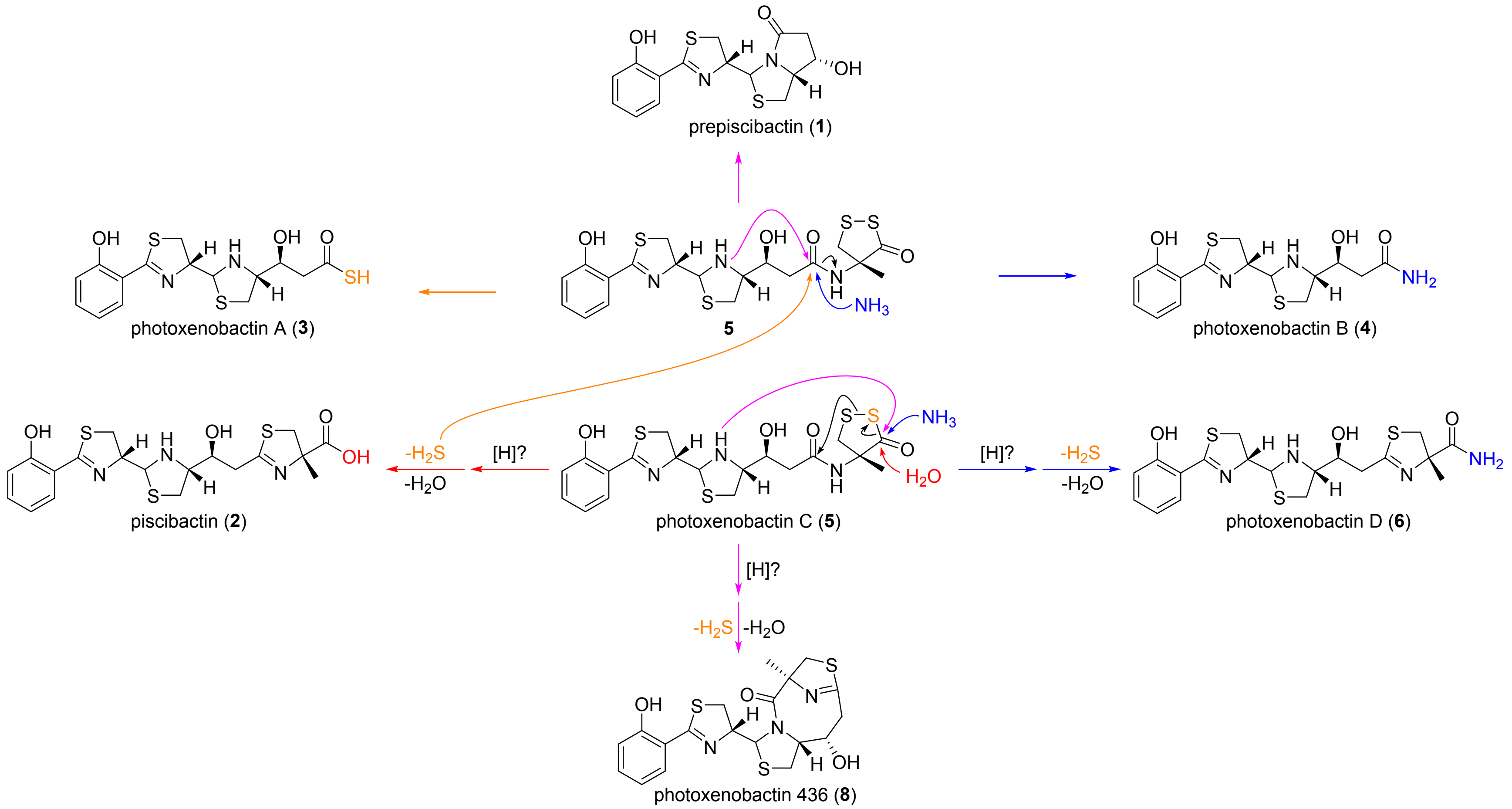

Figure S7. Proposed mechanisms of post-assembly-line conversions of photoxenobactin C (**5**) to other *pxb*-derived products.

References

(1) Fu, C.; Donovan, W. P.; Shikapwashya-Hasser, O.; Ye, X.; Cole, R. H. Hot Fusion: an efficient method to clone multiple DNA fragments as well as inverted repeats without ligase. *PLoS One* **2015**, *9*, e115318.

(2) Wang, G.; Zhao, Z.; Ke, J.; Engel, Y.; Shi, Y.-M.; Robinson, D.; Bingol, K.; Zhang, Z.; Bowen, B.; Louie, K.; et al. CRAGE enables rapid activation of biosynthetic gene clusters in undomesticated bacteria. *Nat. Microbiol.* **2019**, *4*, 2498-2510.

(3) Gualtieri, M.; Ogier, J.-C.; Pagès, S.; Givaudan, A.; Gaudriault, S. Draft genome sequence and annotation of the entomopathogenic bacterium *Xenorhabdus szentirmaii* strain DSM16338. *Genome Announc.* **2014**, *2*, e00190-00114.

(4) Shi, Y.-M.; Hirschmann, M.; Shi, Y.-N.; Ahmed, S.; Abebew, D.; Tobias, N. J.; Grün, P.; Crames, J. J.; Pöschel, L.; Kuttenlochner, W.; et al. Global analysis of biosynthetic gene clusters reveals conserved and unique natural products in entomopathogenic nematode-symbiotic bacteria. *Nat. Chem.* **2022**, *14*, 701-712.

(5) Nollmann, F. I.; Heinrich, A. K.; Brachmann, A. O.; Morisseau, C.; Mukherjee, K.; Casanova-Torres, A. M.; Strobl, F.; Kleinhans, D.; Kinski, S.; Schultz, K.; et al. A *Photorhabdus* natural product inhibits insect juvenile hormone epoxide hydrolase. *ChemBioChem* **2015**, *16*, 766-771.

(6) Bode, E.; Heinrich, A. K.; Hirschmann, M.; Abebew, D.; Shi, Y.-N.; Vo, T. D.; Wesche, F.; Shi, Y.-M.; Grün, P.; Simonyi, S.; et al. Promoter activation in *Δhfq* mutants as an efficient tool for specialized metabolite production enabling direct bioactivity testing. *Angew. Chem. Int. Ed.* **2019**, *58*, 18957-18963.
